# An investigation on the status of resilience amongst adults with blindness in Addis Ababa

**DOI:** 10.4102/ajod.v9i0.628

**Published:** 2020-11-10

**Authors:** Tsigie G. Zegeye

**Affiliations:** 1 College of Education and Behavioral Sciences, Bahir Dar University, Bahir Dar, Ethiopia

**Keywords:** resilience, adults, blindness, demographics, quality of life

## Abstract

**Background:**

Living with blindness for anyone, whether educated or uneducated, rich or poor, with adequate support or without it is seriously limiting. The quality of life of people with blindness is significantly influenced by the level of resilience they possess. The status of resilience of adults with blindness living in Addis Ababa is not known.

**Objectives:**

Against this backdrop, this study was designed to explore the level of resilience of Adults living with blindness. The influence of some demographics on resilience was also examined.

**Method:**

Survey design was employed to carry out the intended objectives of this stud. Data was collected from a random sample of 220 adults with blindness living in Addis Ababa using Connor-Davidson Resilience Scale. Descriptive statistics, t-test and one way ANOVA followed by Scheffe post hoc comparisons were used to analyse the data.

**Results:**

The results revealed that the level of resilience of adults with blindness was found below the average score with a mean score of 46.11. Participants’ gender, time of onset of blindness, marital status and education seemed to influence resilience of blind adults.

**Conclusion:**

Adults having blindness currently living in Addis Ababa are less resilient than needed. Resilience of adults with blindness is differentiated by their demographic characteristics. These people need an integrated effort to enhance their resilience capacity by reducing the barriers and challenges they encounter and promoting protective resources through the different wings of disability related services.

## Introduction

Living with blindness is invariably a challenge to anyone regardless of one’s circumstances. How one lives without significant vision depends upon a plethora of factors within and outside of the person. The living situations of persons with blindness can be comprehended on a continuum where one end characterises utter poverty and almost non-human existence, and the other end portrays a successful life. People with blindness, though significantly less in number, at the positive end of the continuum are considered as resilient persons (Demmitt 2017; Masten & Coatsworth 1998; Waters 2013; Wortaw & Shiferaw 2015), whilst it is automatic that people at the other end are considered less or not resilient ones (Alvord & Grados 2005; Bradley & Corwyn 2002).

Why are some persons with blindness resilient whilst the majority of persons having blindness are not? Although answering this question is not the purpose of this study, the answer to this question takes us to the barriers and challenges as well as protective factors operating on persons with blindness. Whilst protective factors facilitate resilience, barriers and challenges impede resilience (Zolkoski & Bullock 2012).

There are multiple definitions of resilience. According to Luthar (2006), resilience is defined as the ability or set of qualities of people to withstand personal vulnerabilities and rise above environmental adversities. Implied within this definition are three basic canons: (1) resilience resources are static traits found within the individual, (2) exposure to significant adversity or risk and (3) positive adaptation or outcome (Howard, Dryden & Johnson 1999). For Howard et al. (1999), resilience is only a result of personal qualities of the individual. According to these authors, if persons with blindness lack resilience, they will be blamed for not having some internal resilient abilities. However, Daniel and Wassell (2002) argued that resilience can be cultivated at any point during a lifespan and is not considered an inherent trait or characteristic of an individual. This implies that what we call resilience can be made and remade through the continuous and intricate interaction of individual and environmental factors across the lifespan of the person.

Other definitions of resilience acknowledge that resilience is a process that occurs in a distinct context, such as when a person exceeds the expectation that is warranted by an individual’s (or community’s) biographical field (Arrington & Wilson 2000) or a dynamic process through which positive outcomes are achieved in the context of adversity (Cicchetti & Curtis 2007; Masten & Reed 2002). The inclusion of context and process in this definition indicates a recognition that risks and positive outcomes vary between contexts and that resilience occurs as a process over time. These authors, however, did not recognise the fact that every individual including those with blindness can be resilient given the appropriate protective resources. Thus, resilience is not only dependent on the characteristics of the individual, but it is also greatly influenced by processes and interactions arising from the family and the wider environment (Bronfenbrenner 1986).

More recently, Ungar (2008) defined resilience as the outcome from negotiations between people and their environments for the resources to define themselves as successful amidst conditions collectively viewed as adverse. This definition is more comprehensive than preceding definitions as it acknowledges how the individual perceives their environment as they negotiate with it, the protective resources that are available to them in the environment and their own abilities as well as the risk factors or adversities found in the environment. So, it is more useful to think about resilience as residing in the contexts in which people live in and that it exists in people and in relationships between and amongst people (McMahon 2007). Norman (2000) also supported the view of the contextual or relational nature of resilience with his contention that a resilient or adaptive outcome is a process of interaction between environmental and personal factors.

Generally, across research and practice, there has been considerable debate over the definition and operationalisation of resilience (Luthar, Cicchetti & Becker 2000). Some scholars categorised resilience as a process, whilst others view it as an individual trait, some others categorise it as a dynamic developmental process and still some others equate it as an outcome. Within this research project, it is believed that in the context of disability in general and blindness in particular, resilience is best defined as an outcome of successful adaptation to risk situations, provided that characteristics of the individual and environmental situations identify resilient processes. Hence, knowing the status of resilience of persons with blindness is very important, as it informs any education, training and rehabilitation efforts for these individuals.

Research into resilience of typical population has progressively increased over the last five decades. Indeed, when it comes to the experience of disability, resilience is implied and is generally understood to mean an attribute of the individual (Ellis 2013). As a result, adults with disabilities are too often excluded from the category of resilient people simply because they have impairments and hence cannot be resilient (Runswick-Cole & Goodley 2013) and are relegated to the category of the vulnerable and the passive (Goldstein & Brooks 2013). However, as resilience theory has progressed, resilience is no longer considered as existing exclusively within the domain of an individual’s personal qualities and it is found out that support services are also vital in fostering resilience (Ellis 2013). Studies also revealed that resilience develops through the complex interchange between an individual and his or her environment, in which the individual can impact a successful result by utilising internal and external protective factors (Luthar & Cicchetti 2000; Richardson 2002). Hence, resilience can be understood as an aggregate effect of various protective resources that enable a person despite adversity (Campbell-Sills, Cohan & Stein 2006). These insights imply that resilience can be made and remade by forces within and outside of the individual. It is then possible to think that people with disabilities in general and persons with blindness, in particular, can be resilient if we consider resilience not as an individual trait but as a relational product. Hence, it can be argued that building resilience cannot only be a matter of building individual capacity, but also a matter of challenging social, attitudinal and structural barriers that threaten resilience development in the lives of adults with visual disabilities (Katherine, Dan & Rebecca 2014).

Resilience research that was conducted based on the perspectives of people with disabilities in general and those with blindness in particular are disappointingly lacking (Hart et al. 2013). Luthar and Zelazo (2003) also indicated that one of the weaknesses of research on resilience has been the exclusive focus on children and adolescents without disability. As factors that operate on persons with blindness are quite specific to the context in which they live to a great extent, information on the level of resilience of persons with blindness from other contexts would not be of much help for any remedial measures to be initiated to support these individuals having blindness in Ethiopia. However, so far no study has been reported to investigate the level of resilience of adults with blindness (AWB) in the context of Ethiopia. Thus, this study stands be the pioneer one in investigating into the status of resilience amongst AWB in Ethiopia.

The levels of resilience may differ based on one’s developmental stage, gender, onset of blindness, education and marital status. Experiencing blindness before or sometime after birth may not have the same impact on resilience development. Children, adolescents and adults, men and women, the rich and the poor, non-educated and educated as well as those who are married or single may also not have the same level of resilience development (Southwick & Bonanno 2014; Wagnild 2003). Hence, studying resilience of AWB as per these variables would be very influential in designing and implementing appropriate resilience building intervention programmes.

### Rationale and objectives

Ethiopia has one of the world’s highest rates of blindness because of low socio-economic status, low awareness and inadequate health infrastructures (Tirussew 2005; Wortaw & Shiferaw 2015). Being the capital city, Addis Ababa hosts a large number of persons with blindness. This is largely because people with blindness from different regions migrate to Addis Ababa for various reasons, such as seeking service and support, as well as for begging on the streets and religious places. Amongst people with blindness living in Addis Ababa, adults make a significant portion (Yemane, Alemayehu & Abebe 2006). It goes without saying that the plight of persons with blindness, especially adults, in general in Ethiopia is pitiable. Even though the existing local and international legislations grant people with disabilities the right to appropriate and relevant support (e.g. FDRE Constitution 1987; MoE 1994), the available evidences (e.g. Breazeale 2014; Sida 2014; Tirussew 2005) suggest that the situations of the majority of AWB are neither exciting nor encouraging.

What precisely makes the lives of AWB in the context of Ethiopia so deplorable is not specifically known, though poverty, lack of adaptations and accommodations in the environment, poor-quality service provisions, and so on, are some of the factors implicated by Tirussew (2005). However, resilience that is presumed to play a key role in the lives of AWB has not been investigated into thus far within the context of Ethiopia. To date how the demographics of persons with blindness and the time of onset of their impairment are associated with the resilience capacity of AWB in Ethiopian context has not been inquired into. The above paucity in research on disability-related resilience triggered this inquiry. The knowledge brought about to fill the above gaps would invariably inform any intervention programmes aimed at fostering resilience of adults having blindness through education, rehabilitation, skills training, gainful employment opportunities and other support services. This study, therefore, has aimed at assessing the level of resilience of AWB who live in Addis Ababa. Moreover, in particular, the examination of the association between resilience and gender, education, time of onset of blindness and marital status was also another objective of this investigation.

## Methods

### Design of the study

A survey design was employed to examine the resilience status of blind adults residing in Addis Ababa. This design is appropriate when investigating specific variables of a proposed study and when seeking to discover possible relationships between groups of independent and dependent variables (Brink & Wood 1998). Thus, using this design, the resilience status of blind adults and its associations with some demographics were investigated.

### Sample

Adults with blindness who were active members of the Ethiopian National Association for the Blind (ENAP) living in Addis Ababa constituted the population of this study. In ENAP, there were 3000 (1550 men and 1450 women) active adult members in the year 2018–2019. Of this population, 220 adults (110 men and 110 women) between the ages of 20 and 64 years were selected using stratified random sampling technique.

### Instrument

The Connor–Davidson Resilience Scale (CD-RISC) was the instrument used in the present study. Connor–Davidson Resilience Scale is a psychometrically strong 25-item questionnaire rated on a five-point Likert scale with response alternatives ranging from 0 (not true at all) to 4 (true nearly all the time). Scores are summed up to determine the total resilience score that can range from 0 to 100, where higher scores reflect greater resilience. Connor–Davidson Resilience Scale was reported to have sound psychometric properties with greater reliability and validity compared with other resilience scales (Goins, Gregg & Fiske 2013). It was tested across different groups of respondents for reliability and the results yielded an average Cronbach’s alpha of 0.89 and item-total correlations ranged from 0.30 to 0.70.

A review of studies that used the CD-RISC demonstrated that CD-RISC is a valid instrument for measuring individual’s resilience in a variety of populations, such as large community samples, survivors of various traumas, caregivers of persons with Alzheimer’s disease, adolescents, elders, patients in treatment for PTSD, members of different ethnic groups and cultures and selected professionals or athletic groups (see Connor & Davidson 2003). The English version of CD-RISC was translated to Amharic (the mother tongue of the participants and the national language of Ethiopia) following all the rigours of instrument translation and the Amharic version was used for data collection. An internal consistency reliability check was conducted using the final data collected for the study and the Cronbach’s alpha was found to be 0.92.

### Procedures of data collection

On completion of pre-data collection preparations, three data collectors were recruited and trained on data collection procedures. The training included contents on rapport creation, respecting the respondent, reading the items without exerting an influence on response selection by the participants, entry of data in the instrument, and so on.

The survey was administered in a paper and pencil format with the participant sitting near to the data collector in private settings. As the participants were having blindness, the data collectors read everything in the instrument and made sure that the respondents understood what was read. Participants were informed of the purpose of the survey and the ethical guarantees were in place. Subsequently, the data collectors read item by item, secured the responses of the respondents and entered into the instrument. The participants were provided with the opportunity to ask for any clarification at any point in time during the entire data collection process.

### Data analysis

The data were entered into the Statistical Package for Social Sciences (SPSS) (version 22) software and checked and edited in preparation for quantitative analysis. Descriptive statistics were used to calculate the levels of resilience. Independent samples *t*-test, ANOVA and Scheffe post hoc pairwise comparisons were employed to compare the resilience of participants grouped based on some demographics.

### Ethical consideration

Ethical guidelines were followed while conducting the study. Permission was obtained from the participants to use the information they provided solely for the purpose of this study. Participants were assured that their identities would remain anonymous and would not be used while reporting the results. All participants were oriented to understand their rights to confidentiality and anonymity in the research process and the right to withdraw from the research at any time without reason.

## Results

### Resilience status of adults with blindness

To determine the resilience status of AWB, descriptive statistics were computed. The results are presented in Table 1. This provides an indication of the range, minimum and maximum values, as well as the mean and standard deviation for the resilience scale in which adults scored themselves regarding their resilience status.

**TABLE 1:** Descriptive statistics of resilience of adults with blindness (*n* = 220).

Variable	*N*	Range	Minimum score	Maximum score	Mean	Standard deviation
Resilience of adults	220	59	25	84	46.11	11.91

As shown in Table 1, on a possible score range of 0–100, the sample scored a mean resilience score of 46.11 (SD = 11.91). The scores ranged from 25 to 84. In light of the maximum possible score and the minimum possible score that suggest stronger and weak resilience, respectively, the mean score of the sample can be interpreted as significantly low. The minimum and maximum scores also indicate that there were no outlier scores showing higher resilience but that one or more of the individuals showed a resilience score that fell in the high score range, which points to a higher resilience.

### Relationship between demographics and resilience

Sub-samples were formed based on the sample’s gender, onset of blindness, marital status and education. Independent samples *t*-test, one-way ANOVA and post hoc pairwise comparisons were employed to examine if significant differences existed as a function of the demographics mentioned above. Independent samples *t*-test between men (*M* = 49.45, SD = 13.48) and women (*M* = 42.78, SD = 12.66) yielded a statistically significant mean difference on resilience (*t*[218] = 3.17, *p* < 0.002). To examine if time of onset of blindness influenced resilience, participants were grouped into adults with adventitious (*M* = 48.65, SD = 12.05) and congenital (*M* = 43.05, SD = 11.26) blindness. Independent samples *t*-test revealed significant mean difference between adventitiously and congenitally blind adults (*t*[218] = 2.65, *p* < 0.009) on their resilience.

To examine the relationship between marital status and resilience, three sub-samples were formed, namely single (*M* = 42.61, SD = 11.63), married (*M* = 53.17, SD = 14.35) and divorced (*M* = 41.76, SD = 13.50). One-way ANOVA showed a significant mean difference amongst the three groups (*F*[2,217] = 12.68; *p* < 0.000). Follow-up Scheffe post hoc pairwise comparisons disclosed a significant mean difference between those who were single and married (MD = −10.56, *p* < 0.000) and between married and divorced respondents (MD = 11.41, *p* < 0.001). No significant difference was found between single and divorced respondents (MD = 0.85, *p* > 0.05. To explore if level of education influenced resilience, the sample was sub-grouped into with non-formal education (*M* = 37.83, SD = 10.38), with primary education (*M* = 39.66, SD = 9.63), with secondary education (*M* = 49.02, SD = 12.84) and with tertiary level of education (*M* = 52.11, SD = 11.37). Result of one-way ANOVA indicated a significant mean difference amongst the groups compared (*F*[3,216] = 11.86, *p* < 0.000). Further, Scheffe post hoc comparisons revealed significant mean differences in four out of six comparisons made revealing a general trend that as adults’ level of education increases, their resilience also increases. The results of post hoc comparisons are shown in Table 2.

**TABLE 2:** Results of Scheffe post hoc comparisons across level of education (*n* = 220).

Level of education	Primary	Secondary	Tertiary
Non formal	−1.830	−11.187*	−14.277*
Primary	–	−9.358*	−12.448*
Secondary	–	–	−3.090

* , *p* < 0.000.

## Discussion

### Resilience status of adults with blindness

The mean resilience score of 46.11 on a possible score range of 0–100 is invariably an unwelcome position for any population. Although 100 could be considered ideal, a mean score closer to 100 or significantly above 50, the middle value of the possible score range, would have been an encouraging result. Indicating a weak status of resilience, the sample’s mean score fell just below the median scale value. Persons with blindness would require stronger and higher level of resilience than persons without blindness as the demands placed on them by their sensory limitations as well as environmental barriers would demand extra capacities to tackle the day-to-day demands. This becomes more so in underdeveloped or developing world because inclusive provisions and infrastructure are very much limited, if not non-existent, in such societies.

Why is the level of resilience so low in this population? Though answering this question is beyond the aim of this study, an answer to this question can emerge more meaningful after exploring the status of barriers and challenges encountered as well as protective resources available for persons with blindness. Finding low level of resilience amongst AWB may be a surprising result in a country where disability issues have been addressed for several years predominantly through legal and policy initiatives. This result then implies that working on policy and legal issues as well as broadcasting disability issues alone will not enhance the resilience capacity of persons with blindness unless concerted efforts are made to remove or minimise the various barriers and challenges that these individuals face. In line with this finding, several studies (e.g. Alvord & Grados 2005; Bradley & Corwyn 2002) indicated that individual’s resilience capacity or level of resilience is lower when there is higher exposure to barriers or risk factors and lower levels of protective resources available for people with disability at different levels of the environment.

### Resilience and demographics

Resilience is a complex concept and it is defined differently in the context of individuals, families, organisations, cultures and societies. However, there is a general consensus that the empirical study of resilience needs to be addressed from a multiple level of analysis that includes genetic, developmental, demographic, cultural, economic and social variables (Arrington & Wilson 2000; Daniel & Wassell 2002; Luthar 2006; Southwick & Bonanno 2014). Similar to these perspectives, the present study investigated the limited knowledge regarding the associations between resilience of persons with blindness across their demographic variables such as gender, education, onset of blindness and marital status.

Resilience capacity may vary based on the social and environmental resources available for a person. These resources may not be equally provided or available for men and women across different cultures. In a culture where disability is misperceived and stigmatised, people with disabilities face many deprivations and maltreatments at different levels of their environments. This maltreatment and neglect may be more severe when it comes to women with a disability, especially in less developed nations such as Ethiopia (Tirussew 2005) as gender inequality is the order of the day, even today in such developing nations. As expected, the results of the present study indicated a statistically significant resilience mean difference between men and women, wherein men have greater resilience than women. Findings of previous studies from other contexts on the association between resilience and gender were inconsistent. Whilst some studies indicated the absence of associations between resilience and gender (e.g. Wagnild & Young 1993), several other studies found strong associations where the level of resilience was higher in women than men (e.g. Sun & Stewart 2012). These studies attributed greater resilience of women to the presence of more positive connections of women with parents, teachers, adults in the community and peer relations and autonomy experiences of women than men. In contrast, going along with the current result, Friburg et al. (2005) and Bonanno (2004) reported that men predicted increased likelihood of resilient outcomes than women. Furthermore, Bonanno et al. (2007) observed women as less than half as likely to be resilient as men. All these inconsistent results on the association between resilience and gender appear to inform that resilience development varies across contexts and cultures, based on the availability of protective resources in a specific context at a given point in time. In Ethiopia, although women are respected and protected, they are placed far below than men in social significance. Women in Ethiopia have traditionally been considered as child bearers, home makers and not as contributor to the economic resources of the family and society. Hence, for women in general and women with disabilities in particular, protective resources at different levels of the environment may not be made as available as they are for men. Women with blindness are at double disadvantage for being women and having blindness. The lower social status assigned to women coupled with the stigma and stereotypes attached to disability may be jointly contributing to their lower level of resilience in comparison to men. As this study is not in a position to make such a conclusion based on existing data, further research is indicated.

An additional demographic characteristic that was hypothesised to influence resilience was the time of onset of blindness. Blindness that occurs at birth or shortly afterwards (congenital) and acquired later in life (adventitious) will not have the same impact on resilience development as the psychological and day-to-day demands and challenges generated by the time of onset are drastically different. With this presumption, when adventitiously blind respondents were compared with congenitally blind respondents, a statistically significant difference in resilience emerged wherein persons with adventitious blindness were found to be more resilient than those with congenital blindness. On the association between time of onset of blindness and resilience, the existing literature is very much inconsistent. For instance, Bonanno (2004) explored and compared the level of resilience amongst the sighted, congenitally and adventitiously blind people. The results revealed that people with congenital blindness had higher levels of resilience of the three groups. However, another study, consistent with the result of the present study, revealed that people with adventitious blindness had greater resilience than those with congenital blindness (Zeeshan & Aslam 2013). This may be because individuals with adventitious blindness may retain significant visual memory to profit from descriptions of a visual nature. Even when they retain no visual memory, they still hold the advantage of their previous visual learning, which would motivate them to move about, discover and interact with their environment. They are often more active, curious and better coordinated than people with congenital blindness (Bonanno 2004). Furthermore, the intervention and prevention measures in place in the environment where people having blindness live, although vital for both congenitally and adventitiously blind individuals, are of paramount importance for congenitally blind persons as they are to capitalise on such services available for their day-to-day life because they do not have or retain any visual memory. Provisions aiming to rehabilitate or habilitate AWB in Ethiopia are strikingly inadequate as well as inefficient. Stated otherwise, the protective resources available at various environmental sub-systems in Addis Ababa may be very much inadequate for congenitally blind persons than adventitiously blind. People with blindness living in such societies are expected to face serious challenges, impeding their resilience. This is all the more true for congenitally blind persons. Such an insight looks more grounded in the context in which this study was conducted.

On many measures, married people, on average, do better than those who are divorced or living single. Therefore, being married is a sign of an advantaged state as it is associated with higher earnings, longer lived relationships and lower risk of poverty (Clarke & McKay 2008). Marriage also connects people to other individuals, to social groups (e.g. extended family) and to other social institutions, which are additional sources of social benefit (Waite 1995). This evidence implies that being married serves as a buffer against challenges and adversities associated with all types of disabilities and blindness in particular. The comparison made to examine the influence of marital status on resilience of the population yielded a result supporting the above insight. That is, AWB in a married relationship are more resilient than those adults who remain either single or divorced. Furthermore, single and divorced sub-samples do not differ on their status of resilience. This result shows that living in a marriage relationship mitigates the adverse effects of blindness. It may be argued that marriage may be serving as a source of support for a spouse having blindness by promoting responsiveness for the needs of him or her by a partner and acts as an important protective resource to encourage resilience development. Remaining single and living divorced are two most demanding life experiences in adulthood (Stroebe & Stroebe 1997) and this may be more so for persons with blindness because the support that can be expected from a caring partner cannot be replaced with anyone else. Life for single and divorced AWB who live in Addis Ababa may be more demanding because environmental barriers are in abundance here and support systems for them are at the lowest. The positives of being a married couple, with the poor rehabilitation provisions and varying environmental barriers, should explain the difference observed as a function of marital status of the samples.

Education is one of the greatest contributing factors to resilience development for everyone; true to AWB too. Education helps PWBs to have improved life outcomes by providing various means and opportunities to overcome the challenges and barriers of life. It was also found that educated adults including adults having blindness had higher scores on resilience as compared to less and non-educated adults (e.g. Holland & Schmidt 2015 Levine 2003; Zeeshan & Aslam 2013). Similar to those findings, the present study also came up with a statistically significant difference in the status of resilience amongst AWB with differing levels of education. The general pattern observed in this study is that the level of resilience increased as persons with blindness climbed up the ladder of education. Although an ascending trend could be observed in the resilience status of the respondents grouped under non-formally educated, with primary, secondary and tertiary educated, respondents with primary and secondary education do not differ significantly. Other than this, the trend is evident that the more educated groups of AWB were having significantly higher levels of resilience capacity.

Ample evidences exist in the literature linking higher level of resilience and success to higher level of education (e.g. Levine 2003; Adriance & Shaw 2008; Zeeshan & Aslam 2013). It can be reasonably argued that education promotes personal resilience factors such as self-confidence, higher self-esteem and positive view of the future, higher intelligence, self-regulation, effective coping and problem-solving skills, which in turn, strengthen resilience.

Education may also help AWB to find or create external protective resources at various levels of their environments that otherwise would have been remained absent. It can also be expected that higher level of resilience may be accompanied by many positive life outcomes such as securing a good job, higher level of education, being married and having kids. Generally, present and previous results showed the role education plays in nurturing resilience in PWB. Hence, it has a strong implication to place higher stress on education to all the habilitation and rehabilitation efforts designed for persons having blindness.

## Conclusions and implications

This study sheds light on the status of resilience of AWB living under the present context of Addis Ababa, Ethiopia. Adults having blindness currently living in Addis Ababa are less resilient than needed.

Gender makes a difference to resilience; male AWB are more resilient than female adults. Adults with adventitious blindness are more resilient than those with congenital blindness, indicative of the influence played by the time of onset of blindness in resilience development. Being in a marital relationship enhances resilience, blind adults who live in a married relationship are more resilient than those adults who are single and divorced. Education enhances resilience of adults having blindness; those who have higher level of education are more resilient than those with lower levels of education.

The findings of the present study highlight that resilience is differentiated by demographic characteristics of people with blindness, and thus they are subject to change. This has strong implication for all the habilitation and rehabilitation efforts. Helping persons with blindness to live as independently and productively as possible in society is the ultimate objective of any rehabilitation and habilitation efforts, where the philosophy of quality of life underpins such efforts. Quality of life can be achieved only if resilience of persons living with blindness is enhanced. As barriers and challenges at different environmental levels impede resiliency, reducing or eliminating these factors to the fullest extent possible would go a long way in strengthening resilience.

The findings of the present study also highlight the importance of providing persons with blindness with the opportunity for education as education plays a vital role in resilience development. An integrated effort to enhance resilience by reducing the barriers and challenges and promoting protective resources through the different wings of disability-related services so as to build an inclusive society is the pertinent implication of this study. Adults with blindness who live and operate in such a society would naturally be more resilient which, in turn, would enhance the quality of their lives.
